# Effect of flurbiprofen axetil on postoperative delirium for elderly patients

**DOI:** 10.1002/brb3.1290

**Published:** 2019-04-21

**Authors:** Xifan Wang, Yu Wang, Yanan Hu, Liping Wang, Wenshuai Zhao, Lanying Wei, Hong Chen, Fei Han

**Affiliations:** ^1^ Department of Anesthesiology The Third Affiliated Hospital, Harbin Medical University Harbin Heilongjiang China

**Keywords:** elder, flurbiprofen axetil, patient‐controlled analgesia, postoperative delirium

## Abstract

**Objectives:**

Proinflammatory cytokines triggered by surgery and postoperative pain are major causes of postoperative delirium (POD). This study investigated the effects of flurbiprofen axetil on POD when used for postoperative analgesia after major noncardiac surgery in elderly patients.

**Methods:**

Patients over 65 years old were randomly divided into two groups: the sufentanil group (S group), in which 150 μg of sufentanil was used in the patient‐controlled analgesia (PCA) pump for 3 days; the sufentanil combined with flurbiprofen axetil group (SF group), in which 150 μg of sufentanil was combined with 300 mg of flurbiprofen axetil in the PCA pump for 3 days. The Confusion Assessment Method scale was used for POD evaluation. The pain intensity, side effects, and risk factors (age, gender, surgical position, and category of surgery) for POD were evaluated.

**Results:**

Ultimately, 140 patients were included. The overall incidence of POD was not significantly different between the S and SF groups. The incidence of POD was significantly lower in the SF group than in the S group among patients over 70 years (5.1% vs. 20.7%, *p* = 0.045, odds ratio = 0.146, 95% confidence interval = 0.020–1.041). The incidence of POD was no difference in patients classified by the category of surgery, surgical position, or gender between groups. Sufentanil and flurbiprofen axetil in the PCA pump was completely used within 72 hr. The pain intensity, consumed sufentanil dosage of the PCA, and the side effects was not different between groups.

**Conclusions:**

Flurbiprofen axetil might reduce POD in patients over 70 years undergoing major noncardiac surgery.

## 1 INTRODUCTION

Postoperative delirium (POD) is common postoperative complication in elderly patients. POD is associated with increased morbidity, mortality, and costs (Abelha et al., 2013). The incidence of POD was found to be 3.6%–28.3% in elderly patients after noncardiac surgery (Su et al., 2015). A number of factors, age, gender, social status, cognitive impairment, major surgery, anesthesia, comorbidities (cardiovascular and metabolic disease), and hypothermia, are associated with POD, but the exact cause of these conditions has not been confirmed (Kazmierski, Banys, Latek, Bourke, & Jaszewski, 2013; Li, Shao, Zeng, & Liang, 2001). Preoperative routine use of benzodiazepines for the prevention of POD is not recommended unless the patient is extremely anxious or in alcohol withdrawal (Aldecoa et al., 2017; Vliet, Mast, Broek, Westendorp, & Craen, 2012).

An increasing amount of evidence has indicated that surgery triggers the release of proinflammatory cytokines (typically TNF‐α, IL‐1β, IL‐6, and IL‐8), which damage synapses and neurons and ultimately lead to POD via vagal afferents and by crossing the blood‐brain barrier (Aldecoa et al., 2017; Kazmierski et al., 2014). It was reported that parecoxib sodium, a nonsteroidal anti‐inflammatory drug (NSAID) and selective cyclooxygenase (COX)‐2 inhibitor commonly used for postoperative analgesia, reduced the incidence of POD in elderly patients (Mu et al., 2006). Flurbiprofen axetil, another potent NSAID and nonselective COX inhibitor, was used for postoperative analgesia and reduced postoperative opioid consumption (Geng et al., 2015). Administration of flurbiprofen axetil was proved to reduce inflammatory responses and induce neuroprotective effects (Wang et al., 2008; Xu, Tan, Chen, Lou, & Chen, 2008). In this study, we sought to investigate the effects of flurbiprofen axetil on POD when used for postoperative analgesia in elderly patients after major noncardiac surgery.

## 2 MATERIAL AND METHODS

This study was a single‐center, prospective, randomized, double‐blinded study conducted in the department of anesthesiology of the Third Affiliated Hospital, Harbin Medical University. The study procedures were approved by the Ethics Committee of the Third Affiliated Hospital, Harbin Medical University (KY2017‐01) and written informed consent was obtained from all subjects participating in the trial. The trial was registered prior to patient enrollment at Chinese Clinical Trial Registry (ChiCTR‐IPR‐17010845, Principal investigator: Fei Han, Date of registration: 2017‐March‐12). Patients over 65 years who were classified with the American Society of Anesthesiologists (ASA) physical status I‐III and were undergoing selective major noncardiac surgeries (thoracic, general, genitourinary, gynecologic, and orthopedic surgeries) were involved in this study. The exclusion criteria were as follows: regular use of opioids, NSAIDs, sedative, antidepressant or anxiolytic drugs prior to the surgery; drug addiction; severe visual and hearing disorders; preoperative history of schizophrenia, epilepsy, Parkinsonism, or myasthenia gravis; inability to communicate during the preoperative period (coma, profound dementia, or language barriers); brain injury or history of neurosurgery; serious hepatic dysfunction (Child‐Pugh class C); serious renal dysfunction (undergoing dialysis before surgery); allergy to opioid analgesics or NSAIDs; any contraindications to the use of NSAIDs; and preoperative Mini‐Mental State Examination (MMSE) score assessed at one day before surgery less than 17 for illiterate patients (uneducated), less than 20 for elementary‐educated patients (education ≤6 years), and less than 24 for patients with secondary education or above (education >6 years). The age, height, weight, gender, comorbidity, and baseline vital signs of each patient were recorded.

### 2.1 Randomization and masking

According to a random number table, patients were allocated into a sufentanil group (S group) or a sufentanil combined with flurbiprofen axetil group (SF group) at ratio of 1:1. Participants, care providers, and physicians who completed the preoperative and postoperative assessments were blinded to the group assignments throughout the study period until follow‐up was completed for final analysis. In case of an emergency (unexpected or rapid deterioration in the patient's clinical status), physicians were allowed to request unmasking the treatment assignment or adjust or interrupt the study if necessary.

### 2.2 Anesthesia and analgesia procedures

On arrival at the operating room, patients were monitored with electrocardiograms, blood pressure measurements, and pulse oximetry. General anesthesia was induced with sufentanil 0.5 μg/kg (Renfu Pharmaceutical Co., Yichang, China), propofol 1–1.5 mg/kg, and cisatracurium 0.15 mg/kg. Endotracheal intubation was facilitated after 3 min of cisatracurium administration and was connected to a ventilator. Propofol 4–10 mg kg^−1^ hr^−1^ and remifentanil 5–20 μg kg^−1^ hr^−1^ were administered and adjusted according to the hemodynamic responses to surgical stimuli and to maintain a bispectral index between 40 and 60 under anesthesia. The neuromuscular blockade was maintained by intermittent injections of 0.05 mg/kg cisatracurium as needed. The ventilation rate was 12/min. Tidal volume was adjusted to 6–10 ml/kg to maintain the end‐tidal CO_2_ level at 35–45 mmHg.

All patients in the S and SF groups received patient‐controlled analgesia (PCA) after surgery. The PCA pump of the S group was filled with 0.5 μg/ml sufentanil (150 μg sufentanil in 300 ml of 0.9% saline). The PCA pump of the SF group was filled with 0.5 μg/ml sufentanil combined with 1 mg/ml flurbiprofen axetil (Taide, Pharmaceutical Co., Beijing, China, 150 μg sufentanil and 300 mg flurbiprofen axetil in 300 ml of 0.9% saline). The PCA protocol was set up as a continuous infusion dose of 4 ml/hr, a bolus dose of 3 ml if needed, and a lock‐out time of 15 min in both groups. The PCA pump in both groups was used for up to 72 hr after surgery, until all of the solution ran out. No refilling of the PCA pump in either group was allowed by the study protocol. Acute rescue analgesic drugs, namely, tramadol (100 mg i.v.), were used when the visual analog scale (VAS: 0, no pain; 10, severe pain) score was more than 4 after three continuous bolus infusions of PCA. If severe and persistent postoperative nausea, vomiting, headache, dizziness, and hypotension (confirmed as less than 30% of the baseline), respiratory depression, gastrointestinal blood or renal dysfunction occurred, the PCA would be stopped, and the patients were switched to an alternate analgesic modality and excluded from the study.

### 2.3 Measurements

The primary endpoint of this study was the incidence of POD on postoperative days 1, 2, 3, and 7 in both groups. POD was diagnosed using the Confusion Assessment Method (CAM) scale. CAM scores consist of four features: (a) acute and fluctuating changes in mental status, (b) inattention, (c) disorganized or incoherent thinking, and (d) an altered concentration of consciousness. Delirium is considered if the patient displayed acute onset of mental status changes or fluctuating course and inattention, with either disorganized thinking or altered level of consciousness. The assessment of POD was performed on postoperative days 1, 2, 3, and 7 by a same physician who was blinded to the group assignments. Prior to the study, the physician was trained according to previous studies (Avidan et al., 2014; Ely et al., 2001). The physician was considered qualified until the CAM assessment of two delirious and two non‐delirious patients he evaluated met the assent of the delirium expert.

The secondary endpoints included postoperative pain intensity at rest and with movement (evaluated by VAS score on postoperative days 1, 2, 3, and 7), the consumed sufentanil dosage of PCA, and the number of PCA attempts during the first day after surgery. The sedation level was assessed on postoperative days 1, 2, 3, and 7 using the Observer's Assessment of Alertness/Sedation Scale (OAA/S: 0, no response to painful stimuli; 5, responds readily to name spoken in a normal tone) and the Ramsay sedation score (RSS: 0, not quiet, irritable; 5, deep sleep, call wake up). Factors such as age, gender, surgical position (lateral vs. supine), and category of surgery (minimally invasive vs. open) that contributed to POD were analyzed between the two groups. Duration of anesthesia and surgery, blood loss, blood transfusion, fluid volume administered during surgery, and side effects (nausea, vomiting, headache, dizziness, hypotension, respiratory depression, gastrointestinal blood, and renal dysfunction), were compared between two groups.

### 2.4 Sample size

Based on an expected incidence of the primary endpoint as a 16.7% occurrence of POD in the S group and 6.7% in the SF group in our preliminary experiments, the minimum sample size was calculated. For a two‐sided difference with 80% power at the 0.05 significance level (*α* = 0.05, *β* = 0.20), 64 participants in each group were required.

### 2.5 Statistical analysis

The data were imported into SPSS (18.0, Chicago, IL) for analysis. Normally distributed data are expressed as the mean ± *SD*. Level variables and abnormally distributed data are presented as the median with interquartile range. The incidence of POD, gender, ASA physical status, education level, site of surgery, blood transfusion, side effects and between the S group and the SF group were compared using a chi‐squared test or Fisher's exact probability test. The general characteristics of the patients, including age, height, weight, duration of anesthesia and surgery, blood loss, the fluid volume, and sufentanil dosage of the PCA on postoperative 1 day, were analyzed via ANOVA followed by the Student‐Newman‐Keuls test. The characteristics of the patients over 70 years, gender, ASA physical status, educational level, site of surgery, and surgical position, associated with POD between the two groups were analyzed via a Binary Logistic Regression analysis. The odds ratio (OR) values and 95% confidence interval (CI) were presented. The VAS scores, OAA/S scores, RSS scores, and the number of PCA attempts on postoperative day 1 were analyzed using a Kruskal‐Wallis H test. A value of *p* < 0.05 was considered statistically significant.

## 3 RESULTS

### 3.1 Patient characteristics

**Figure 1:**
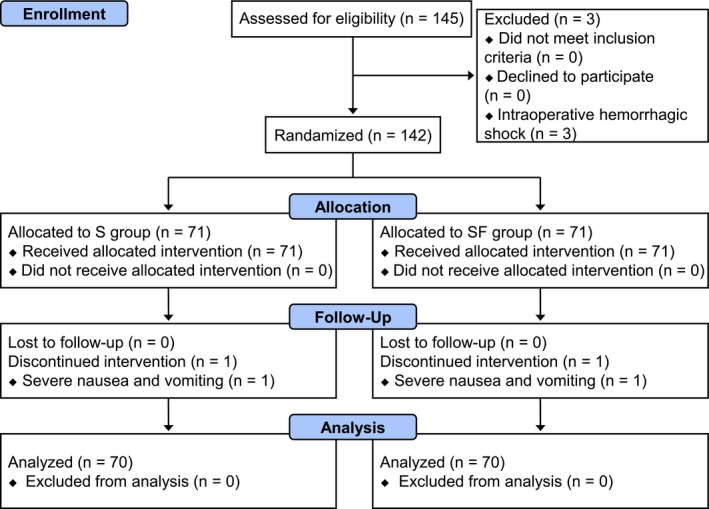
CONSORT diagram. S group: sufentanil group; SF group: sufentanil combined with flurbiprofen axetil group

Between Mar 13, 2017 and Nov 5, 2017, 145 patients were involved in this study. Three patients who suffered unexpected intraoperative hemorrhagic shock were excluded. In each group, there was one case of discontinued intervention due to severe nausea and vomiting. Ultimately, 140 patients were included in the analysis (Figure 1). There were no differences in the general characteristics (age, height, weight, gender, ASA physical status, education level before surgery, and MMSE score before surgery) of the patients, site of surgery, duration of anesthesia and surgery, blood loss, blood transfusion, or fluid volume between the two groups (Table 1).

**Table 1:** Baseline and surgical characteristics were no difference between the two groups

Characteristics	S group (*n* = 70)	SF group (*n* = 70)	*p* value
Age, years	69.3 ± 4.6	69.5 ± 4.1	0.833
Age groups, *n* (%)			0.128
65–69 years	41 (58.6%)	31 (44.3%)	
≥70 years	29 (41.4%)	39 (55.7%)	
Height, cm	165.7 ± 8.3	164.2 ± 7.8	0.292
Weight, kg	64.6 ± 7.4	65.1 ± 9.7	0.736
Gender, male (%)	29 (58.6)	34 (48.6)	0.396
ASA physical status, *n* (%)			0.645
I	8 (11.4%)	10 (14.3%)	
II	58 (82.9%)	57 (81.4%)	
III	4 (5.7%)	3 (4.3%)	
Education, *n* (%)			0.469
≤9 years	62 (88.6%)	58 (82.9%)	
>9 years	8 (11.4%)	12 (17.1%)	
MMSE score before surgery	26.3 ± 2.8	26.2 ± 2.5	0.284
Site of surgery, *n* (%)			0.544
Intra‐thoracic	14 (20%)	19 (27.1%)	
Intra‐abdominal	48 (68.6%)	42 (60.0%)	
Others	8 (11.4%)	9 (12.9%)	
Duration of anesthesia, h	3.7 ± 1.2	3.7 ± 1.2	0.904
Duration of surgery, h	2.7 ± 1.1	2.8 ± 1.2	0.848
Blood loss, ml	166.0 ± 143.3	156.3 ± 128.1	0.479
Blood transfusion, *n* (%)	7 (10%)	7 (10%)	1.000
Fluid volume, ml	1732.8 ± 526.5	1787.5 ± 638.8	0.354

Abbreviations: ASA: American Society of Anesthesiologists; MMSE: mini‐mental state examination; S group: sufentanil group; SF group: sufentanil combined with flurbiprofen axetil group.

### 3.2 Outcome evaluation

**Figure 2:**
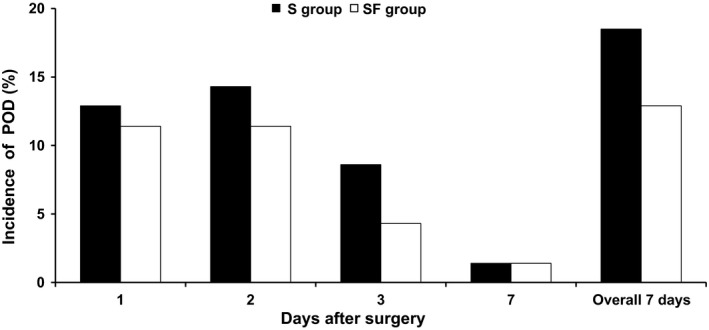
Incidence of POD in the two groups on postoperative days 1, 2, 3, and 7. POD: postoperative delirium; S group: sufentanil group; SF group: sufentanil combined with flurbiprofen axetil group

**Figure 3:**
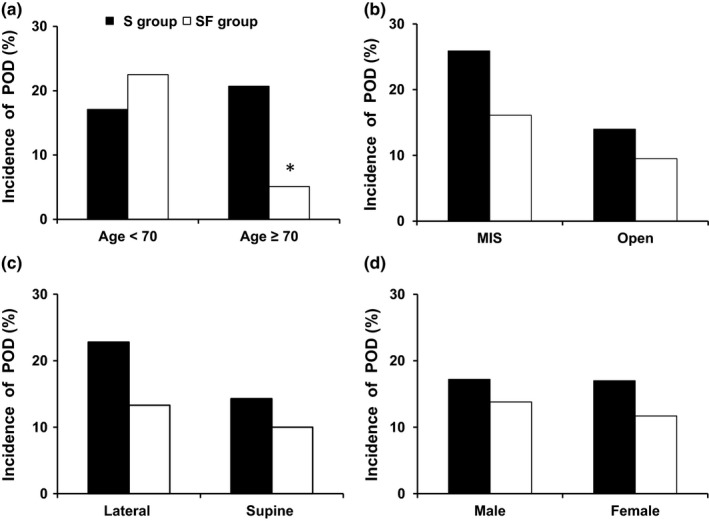
Incidence of POD between the two groups in patients aged over 70 years or not (a), patients undergoing MIS or open surgery (b), patients with lateral or supine position (c), and patients of different genders (d) during the overall 7 days. *p < 0.05, the S group compared with the SF group. MIS: minimally invasive surgery; POD: postoperative delirium; S group: sufentanil group; SF group: sufentanil combined with flurbiprofen axetil group

The overall incidence of POD was not significantly different between the S group (13 of 70, 18.6%) and the SF group (9 of 70, 12.9%, Figure 2). The incidence of POD was significantly lower in the SF group (2 of 39, 5.1%) than in the S group (6 of 29, 20.7%) in patients over 70 years (*p* = 0.041, Figure 3). Flurbiprofen axetil significantly reduced POD in patients over 70 years (*p* = 0.045, OR = 0.146, 95% CI = 0.020–1.041). The other factors in patients over 70 years, gender, ASA physical status, education level, site of surgery, and surgical position, associated with POD between the two groups were no statistical difference (Table 2). The incidence of POD was no difference in patients classified by the category of surgery, surgical position, or gender between the two groups.

**Table 2:** Factors associated with POD in patients over 70 years in the two groups

Characteristics	S group	SF group	OR	95% CI	*p* value
Age, years	74.2 ± 3.3	73.5 ± 2.8	0.237	0.053–7.316	0.439
Gender, male (%)	17 (58.6%)	14 (35.9%)	0.597	0.076–4.687	
ASA physical status, *n* (%)			7.727	0.657–90.819	0.104
II	26 (89.7%)	37 (94.8%)			
III	3 (10.3%)	2 (5.1%)			
Education, *n* (%)			3.239	0.276–37.970	0.349
≤9 years	25 (86.2%)	33 (84.6%)			
>9 years	4 (13.8%)	6 (15.4%)			
Site of surgery, *n* (%)			0.679	0.021–4.905	0.413
Intra‐thoracic	8 (27.6%)	11 (28.2%)			
Intra‐abdominal	21 (72.4%)	28 (71.8%)			
Others	0 (0%)	0 (0%)			
Surgical position, *n* (%)			8.619	0.679–109.476	0.097
Lateral	10 (34.5%)	15 (38.5%)			
Supine	19 (65.5%)	24 (61.5%)			
Others	0 (0%)	0 (0%)			

Abbreviations: ASA: American Society of Anesthesiologists; CI: confidence interval; OR: odds ratio; POD: postoperative delirium; S group: sufentanil group; SF group: sufentanil combined with flurbiprofen axetil group.

VAS scores at rest or with movement were not different between the two groups on postoperative days 1, 2, 3, or 7 (Table 3). OAA/S scores and RSS scores were no different between the two groups. There were no differences in postoperative nausea, vomiting, headache, dizziness, hypotension, respiratory depression, gastrointestinal blood, or renal dysfunction between the two groups. The consumed flurbiprofen axetil dosage of PCA on postoperative day 1 in the SF group was 94.1 ± 39.5 mg. The consumed sufentanil dosage of PCA on postoperative day 1 was no different between the S group (52.2 ± 13.7 μg) and the SF group (47.0 ± 19.7 μg). The number of PCA attempts on postoperative day 1 was no different between the S group and the SF group (1.5 ± 2.7 vs. 1.2 ± 3.0). The consumed flurbiprofen axetil dosage of PCA for 3 days was 0 mg in the S group and 300 mg in the SF group. The consumed sufentanil dosage of PCA for 3 days was no different between the S group and the SF group (150 μg in each group). No acute rescue analgesic drugs were needed in the S group or the SF group.

**Table 3:** Postoperative pain intensity, sedation, the usage of PCA, and postoperative complications in the two groups

Characteristics	S group (*n* = 70)			SF group (*n* = 70)	*p* value
VAS score at rest					
Day 1 after surgery	1 (0,3)			1 (0,3)	0.977
Day 2 after surgery	0 (0,2)			0 (0,2)	0.946
Day 3 after surgery	0 (0,0)			0 (0,0)	0.321
Day 7 after surgery	0 (0,0)			0 (0,0)	1.000
VAS score with movement					
Day 1 after surgery	3 (0,5)			3 (0,5)	0.465
Day 2 after surgery	2 (0,4)			2 (0,5)	0.289
Day 3 after surgery	0 (0,3)			0 (0,4)	0.816
Day 7 after surgery	0 (0,0)			0 (0,0)	1.000
OAA/S score					
Day 1 after surgery	5 (5,5)			5 (5,5)	0.317
Day 2 after surgery	5 (5,5)			5 (5,5)	1.000
Day 3 after surgery	5 (5,5)			5 (5,5)	1.000
Day 7 after surgery	5 (5,5)			5 (5,5)	1.000
RSS score					
Day 1 after surgery	2 (2,2)			2 (2,2)	1.000
Day 2 after surgery	2 (2,2)			2 (2,2)	1.000
Day 3 after surgery	2 (2,2)			2 (2,2)	1.000
Day 7 after surgery	2 (2,2)			2 (2,2)	1.000
PCA pump at day 1 after surgery					
Flurbiprofen consumption, mg	0			94.1 ± 39.5	0.000
Sufentanil consumption, μg	52.2 ± 13.7			47.0 ± 19.7	0.074
PCA attempts, *n*	1.5 ± 2.7			1.2 ± 3.0	0.370
Postoperative complications, *n* (%)					
Nausea and vomiting	8 (11.4%)			12 (17.1%)	0.334
Headache	2 (2.9%)			4 (5.7%)	0.404
Dizziness	6 (8.6%)			15 (21.4%)	0.099
Hypotension	2 (2.8%)			0 (0%)	0.496
Respiratory depression	0 (0%)			0 (0%)	–
Gastrointestinal blood	0 (0%)			0 (0%)	–
Renal dysfunction	0 (0%)			0 (0%)	–

Abbreviations: OAA/S: observer's assessment of alertness/sedation scale; PCA: patient‐controlled analgesia; RSS: Ramsay sedation score; S group: sufentanil group; SF group: sufentanil combined with flurbiprofen axetil group; VAS: visual analog scale.

## 4 DISCUSSION

To the best of our knowledge, this is the first report to demonstrate the effects of flurbiprofen axetil on POD in elderly patients undergoing major noncardiac surgery. The occurrence of POD was 18.6% in the S group, which were consistent with previous reports (Inouye, Westendorp, & Saczynski, 2014; Su et al., 2015). This study indicated that flurbiprofen axetil reduced POD in patients over 70 years undergoing major surgery. No additional side effects were found when flurbiprofen axetil was used for the PCA. Flurbiprofen axetil as an NSAIDs involving alternative multimodal strategies was widely used for the management of postoperative pain after surgical trauma (Xu et al., 2008). In this study, satisfactory analgesia was achieved in both groups but the sufentanil consumption of PCA in the SF group was not reduced than that in the S group. It was reported that flurbiprofen axetil combined with smaller dosage of fentanyl significantly enhanced analgesia effect and reduced side effects of vomiting (Wen et al., 2015). However, in other studies, postoperative administration of flurbiprofen axetil, 100 mg/day, did not reduce the opioids consumption or improve analgesia effects (Liu, Chai, & Chen, 2011; Nonaka, Hara, Miyamoto, Sugita, & Yamamoto, 2016). In our study, the dosage of flurbiprofen axetil used is the most common dosage in clinical practice for PCA and the results of our study on the consumption of opioids and analgesia is consistent with previous reports (Liu et al., 2011; Nonaka et al., 2016).

A strong association was shown between elevated biomarkers of inflammation and the risk of developing POD. Surgical trauma engages the innate immune system through NF‐κB‐dependent signaling to release cytokines that disrupt blood‐brain barrier integrity. Through a permeable blood‐brain barrier, peripheral macrophages migrate into the hippocampus, thereby promoting neuroinflammation that impairs memory (Terrando et al., 2011). Compelling evidence has shown that acute peripheral inflammatory stimulation induces activation of brain parenchymal cells and expression of proinflammatory cytokines and inflammatory mediators in the CNS, leading to potential cerebral edema, possibly causing POD and cognitive decline (Harten, Scheeren, & Absalom, 2012). Flurbiprofen axetil preconditioning improves neurological function and neuronal survival, which is associated with inhibition of IL‐β, TNF‐α, and TXB2 synthesis (Wu et al., 2018). Flurbiprofen axetil has been found to demonstrate a high affinity for inflammatory tissues due to its lipid microsphere system (Jiang et al., 2015). These findings indicated the potential mechanism of inhibiting COX and proinflammatory cytokine release for COX inhibitors to prevent the occurrence of POD in the brain and CNS as a postoperative analgesic.

Interestingly, in this study, flurbiprofen axetil decreased the incidence of POD in patients over 70 years of age. It should be noted that POD are age‐dependent complications after surgery that involve postoperative reductions in memory, mental flexibility, and information processing (Rasmussen et al., 2001). The probability of transitioning to delirium increases dramatically for each year of life after 65 years (Pandharipande et al., 2006). The aging brain exhibits increased vulnerability due to reduced cholinergic reserves and a high prevalence of cognitive impairment (Ljubisavljevic & Kelly, 2003).

In addition to age, there are many other risk factors induced by POD, such as hypothermia, dehydration, site of surgery (abdominal and cardiothoracic), category of surgery, surgical position, and duration of surgery. In this study, there was a higher number of POD (25.9% vs. 14%) cases in patients undergoing minimally invasive surgery (MIS) than in patients undergoing open surgery, respectively, but this difference did not reach statistical significance. It was indicated that patients undergoing MIS suffered a less incidence of POD and lower inflammatory markers compared with those with open surgeries, consistent with a lower degree of stress response (Tan et al., 2015). However, recent work suggested that intraoperative CO_2_ control may be an independent marker of POD risk (El‐Gabalawy et al., 2017; Mutch & El‐Gabalawy, 2017). Previous studies revealed that mild hypercapnia and substantial vasodilation in the brain induced during MIS resulted in alterations in cerebrovascular reactivity to a CO_2_ stress in patients that may be used to identify patients at cognitive risk (Wang et al., 2017). Evidence is emerging that intraoperative CO_2_ accumulation may be a significant stressor for POD in susceptible individuals, and tight control of end‐tidal CO_2_ around the patient's baseline normocapnic values may be an important modifier that limits POD in adults (Mutch & El‐Gabalawy, 2017).

A variety of studies have confirmed the influence of surgical position on changes in cerebral homeostasis and regional cerebral oxygenation (rScO_2_) that lead to cognitive dysfunction and delirium (Jo, Kim, Lee, Lee, & Kwak, 2016; Salazar et al., 2013). In our study, no differences in POD were found between the lateral position and the supine position. Similarly, Murphy et al. demonstrated that rScO_2_ was maintained during arthroscopic shoulder surgery in the lateral decubitus position (Murphy et al., 2010).

Regarding the association between gender and cognitive function, there is evidence for a female advantage in episodic memory for verbal information and a male advantage in visuospatial memory (Beinhoff, Tumani, Brettschneider, Bittner, & Riepe, 2008). Further analysis revealed that men with a more prevalence of sepsis, coronary artery disease, hyponatremia, and hypokalemia, and women with hypertension, conduction disorders, myocardial infarction, and dementia presented a higher incidence of delirium (Serpytis et al., 2017). However, the frequency of delirium after surgery was similar between women and men in many reports (Hogue, Sundt, Barzilai, Schecthman, & Davila‐Roman, 2001). In a ten‐year study of risk factors for delirium, Zaal et al. concluded that gender was not associated with delirium in critically ill adults undergoing noncardiac surgery (Zaal, Devlin, Peelen, & Slooter, 2015). Similarly in our study, the occurence of delirium was no difference between women and men. The influence of gender on the incidence of POD still requires further study in the future.

The present study had some limitations. First, this study did not detect the variables of inflammatory mediators, although the effect of flurbiprofen axetil on POD was believed to reduce inflammatory responses. However, several studies pointed out that plasma specimens used for assessing proinflammatory cytokines levels were not suitable compared to cerebrospinal fluid samples (Hirsch et al., 2016; Lachno et al., 2013). Second, the duration of delirium was not assessed in this study, although it was an important factor to evaluate POD.

## 5 CONCLUSIONS

Flurbiprofen axetil used for PCA might reduce POD in patients over 70 years undergoing major noncardiac surgery.

## CONFLICTS OF INTEREST

The authors declare that they have no conflicts of interest.

## DATA AVAILABILITY STATEMENT

The data that support the findings of this study are available on request from the corresponding author. The data are not publicly available due to privacy or ethical restrictions.
